# Supplementary material for: Evaluating the accuracy of ChatGPT model versions for giving care-seeking advice
Source: Commun Med (Lond). 2026 Feb 25;6:171. doi: 10.1038/s43856-026-01466-0 (PMC13031804; doi:10.1038/s43856-026-01466-0)
Supplement: Supplementary file 3 — Description of Additional Supplementary Files [file 43856_2026_1466_MOESM3_ESM.docx]

**Description of Additional Supplementary Files**

Supplementary Data 1: The source data for all figures and analyses
